# A life-history perspective on sexual selection in a polygamous species

**DOI:** 10.1186/s12862-020-01618-3

**Published:** 2020-05-07

**Authors:** Ke Gao, Michiel van Wijk, Zoe Clement, Martijn Egas, Astrid T. Groot

**Affiliations:** 1 Institute for Biodiversity and Ecosystem Dynamics, University of Amsterdam, Science Park 904, 1098 XH Amsterdam, Netherlands; 2 Department Entomology, Max Planck Institute for Chemical Ecology, Hans Knoell Strasse 8, 07745 Jena, Germany

## Abstract

**Background:**

Ever since Darwin, evolutionary biologists have studied sexual selection driving differences in appearance and behaviour between males and females. An unchallenged paradigm in such studies is that one sex (usually the male) signals its quality as a mate to the other sex (usually the female), who is choosy in accepting a partner. Here, we hypothesize that in polygamous species these roles change dynamically with the mating status of males and females, depending on direct reproductive costs and benefits of multiple matings, and on sperm competition. We test this hypothesis by assessing fitness costs and benefits of multiple matings in both males and females in a polygamous moth species, as in moths not males but females are the signalers and males are the responders.

**Results:**

We found that multiple matings confer fitness costs and benefits for both sexes. Specifically, the number of matings did not affect the longevity of males or females, but only 67% of the males and 14% of the females mated successfully in all five nights. In addition, the female’s reproductive output increased with multiple matings, although when paired with a new virgin male every night, more than 3 matings decreased her reproductive output, so that the Bateman gradient for females fit a quadratic model better than a linear model. The male’s reproductive success was positively affected by the number of matings and a linear regression line best fit the data. Simulations of the effect of sperm competition showed that increasing last-male paternity increases the steepness of the male Bateman gradient and thus the male’s relative fitness gain from additional mating. Irrespective of last-male paternity value, the female Bateman gradient is steeper than the male one for up to three matings.

**Conclusion:**

Our results suggest that choosiness in moths may well change throughout the mating season, with males being more choosy early in the season and females being more choosy after having mated at least three times. This life-history perspective on the costs and benefits of multiple matings for both sexes sheds new light on sexual selection forces acting on sexual signals and responses.

## Background

Sexual selection studies historically hypothesize that one sex (typically male) signals its quality to the choosy sex (typically female) [1–4]. In polygamous species, however, females as well as males can gain direct benefits from additional matings [5]. In general, the less competitive sex is generally hypothesized to be the more choosy sex [6]. Which sex is more or less competitive depends among others on variance in reproductive success (the sex with the higher variance is more competitive) [7, 8], the amount of investment in reproduction [9] and on how much time each sex spends in sexually unreceptive state, as this affects the operational sex ratio [10–12]. When variance in reproductive success is similar in males and females, mutual mate choice is expected [3, 13].

Multiple matings have in previous decades been assumed to be mostly beneficial to males, who can maximize their fitness by maximizing the number of matings, while the reproductive success of females was assumed to be largely independent of the number of matings [1, 8]. These assumptions are generally known as the “Bateman’s principle” [4, 14]. In the past decade, [4] combined Bateman’s principle with the “ardent male – coy female” hypothesis of [2] to the “Darwin-Bateman paradigm” that integrates the ideas that ‘male variance of reproductive success exceeds that of females’, ‘males have more to gain from multiple matings’, and ‘males are generally ardent and females are generally coy’. However, it is now commonly found that females as well as males can gain direct benefits from additional matings [7, 8], for example through nuptial gifts [15, 16]. To determine the differential benefits to reproductive success to be gained by males and females when mating multiple times, the “Bateman gradient” should be calculated [7], which is the relation between the number of mates and the number of offspring, specifically the least-square regression of reproductive success on mating success [17].

Fitness benefits of multiple matings can be offset by costs. The costs of multiple matings can be in the form of expending physiological energy in offspring, for example through large gametes (females) or large spermatophores (males), or expending behavioral energy through parental care or intrasexual competition, which affect the operational sex ratio (OSR) [3]. The caring or competing sex has a ‘time out’ of the mating pool, leading to an OSR biased towards the uncaring or uncompeting sex [6]. For example, if males show more intrasexual competition than females, and females spend less time on parental care than males spend on competition, the OSR will be biased towards females, which may result in male choice [10, 11]. Thus, the Bateman gradient and the OSR are complementary measures of the strength of sexual selection: while the Bateman gradient is a measure of the fitness gain per mating, the OSR is a measure of the difficulty of achieving a mating [12].

Variance in reproductive success may change in the course of a mating season, depending on direct reproductive costs and benefits of multiple matings, changes in reproductive success over the course of a lifetime, and on sperm competition [18]. For example, early in life virgin females may be less choosy than later in life, i.e., when she is mated, to ensure fertilization of her eggs [19, 20]. In addition, in a number of species, female reproductive success has been found to have an optimum - increasing with up to two to three matings, but decreasing with additional matings [21]. Male reproductive success may be affected by the mating status of the female, because males may lose paternity due to sperm precedence [22, 23]. In addition, male reproductive success may be affected by the costs of producing sperm [24]. As males may be sperm-limited [22, 25], male sperm allocation and refractory period for sperm production between matings may be a trade-off between male investment in current versus future reproduction [24]. Thus, in the course of a mating season, who is the limiting sex may change, either due to a longer refractory period of one of the sexes, which changes the OSR, or because variance in reproductive success changes.

To determine how sexual selection dynamics change over the course of a lifetime in a polygamous moth species, we investigated the direct reproductive costs and benefits of multiple matings, as well as the role of sperm competition in reproductive success. Moths are atypical in the sense that females, not males, are the attractive sex [21], which occurs by emitting a species-specific sex pheromone through which males are attracted from a distance [26, 27]. As males are behaviorally tuned to their species-specific pheromone blend [28–30], a deviation from the mean blend is likely to lower her reproductive fitness [31, 32], which makes it difficult to understand the high diversity of moth pheromone blends found in nature [33–36]. Possibly, changing sexual selection forces during the adult mating life allows for more variation in sexual signals and/or responses than generally assumed.

In this study, we aimed to assess who is the choosy sex in the polygamous moth species *Heliothis virescens* (Lepidoptera, Noctuidae) by determining the fitness costs and benefits of multiple matings. Of this species, much information on its sexual behaviors has been gathered over the past 40 years, because this species is a major pest in North and South America. Specifically, *H. virescens* males and females are highly promiscuous [37–41]. Field-caught females have been found to contain 7 spermatophores [39] and females mate up to 12 times under laboratory conditions [42]. Both males and females can mate only once a night [39, 43], as each mating lasts on average 3 h [43] and adults are sexually active only for 3–5 h during the night [44, 45]. However, both sexes mate multiple nights ([37–41]; this study). The average adult life time is 20 days in the laboratory at 25 °C [46], during which females oviposit ~ 500–1500 eggs ([47]; this study).

The female’s direct investment in reproduction starts by attracting males through ‘calling’, i.e., extruding her sex pheromone gland and emitting a long-range pheromone. This may result in fitness and physiological costs [48, 49] and may make her vulnerable to parasitism and predation [50, 51]. Mate choice experiments indicate that females do not discriminate between virgin or mated males [43].

The male’s direct investment in each mating starts with his searching behavior, which is followed upon arrival by his pheromone display, and by the production of a large spermatophore during mating, which consists not only of eupyrene and apyrene sperm, but also of proteins [41, 42, 52], so that the spermatophore may partly act as a nuptial gift. His investment in searching behavior is hard to quantify, as it strongly depends on moth density and the prevailing operational sex ratio. The cost of both spermatophore and male pheromone production have not been quantified either, but one spermatophore may be up to 10% of the male’s body weight [42].

Sperm precedence and paternity allocation studies in *H. virescens* have shown that paternity patterns are quite variable, ranging from 100% first or last male sperm precedence to complete sperm mixing [37, 38, 40, 41, 52, 53], although on average the last male gains ~ 66% paternity [53]. In mate choice experiments, we recently found that males choose virgin partners over mated partners [43].

Based on all this information, we hypothesized that in this polygamous species the choosing sex roles change dynamically with the mating status of males and females, depending on direct reproductive costs and benefits of multiple matings, and on sperm competition. To identify the factors that may affect reproductive success, we used two different setups: repeated matings and polygamous matings [following 54]. In repeated matings, the same males and females are confined for 5 consecutive nights, while in polygamous matings, each male or female gets a new, virgin mating partner every night. To take different degrees of paternity into account, we also explored differences in absolute reproductive success between males and females using computer simulations.

## Results

To determine the fitness costs and benefits of multiple matings, we assessed longevity and lifetime fecundity and fertility of individual moths by setting up four treatments (see Table 1): a) one virgin male or female was placed per beaker and observed for five consecutive nights (treatment 1), b) virgin individuals were paired with a virgin mate on the first night, after which the sexes were separated into different beakers (treatment 2), c) individual moths were paired with a new, virgin mate every night for five consecutive nights (treatment 3: polygamous matings), d) individual moths were paired with the same mate every night (treatment 4: repeated matings) for five consecutive nights (see Methods for more details on the experimental design). In total, we observed 229 copulations, while we found 225 spermatophores in dissected females, which means that 98.3% of the observed matings were successful copulations. Below we specify the costs and benefits of multiple matings in terms of longevity, number of observed matings in repeated and polygamous matings, reproductive output, and the effects of sperm precedence.

**Table 1:** Experimental treatments with number (n) of females and males tested in each treatment

Treatment number	Name	Description	n females	n males
1	No mate	Individual virgin moths	30	30
2	One mate	One mate on the first night	29	31
3	Different mates (polygamous matings)	Five different mates	59	63
4	Same mates (repeated matings)	Same mate for 5 nights	52	52

### Number of matings do not affect longevity

**Fig. 1:**
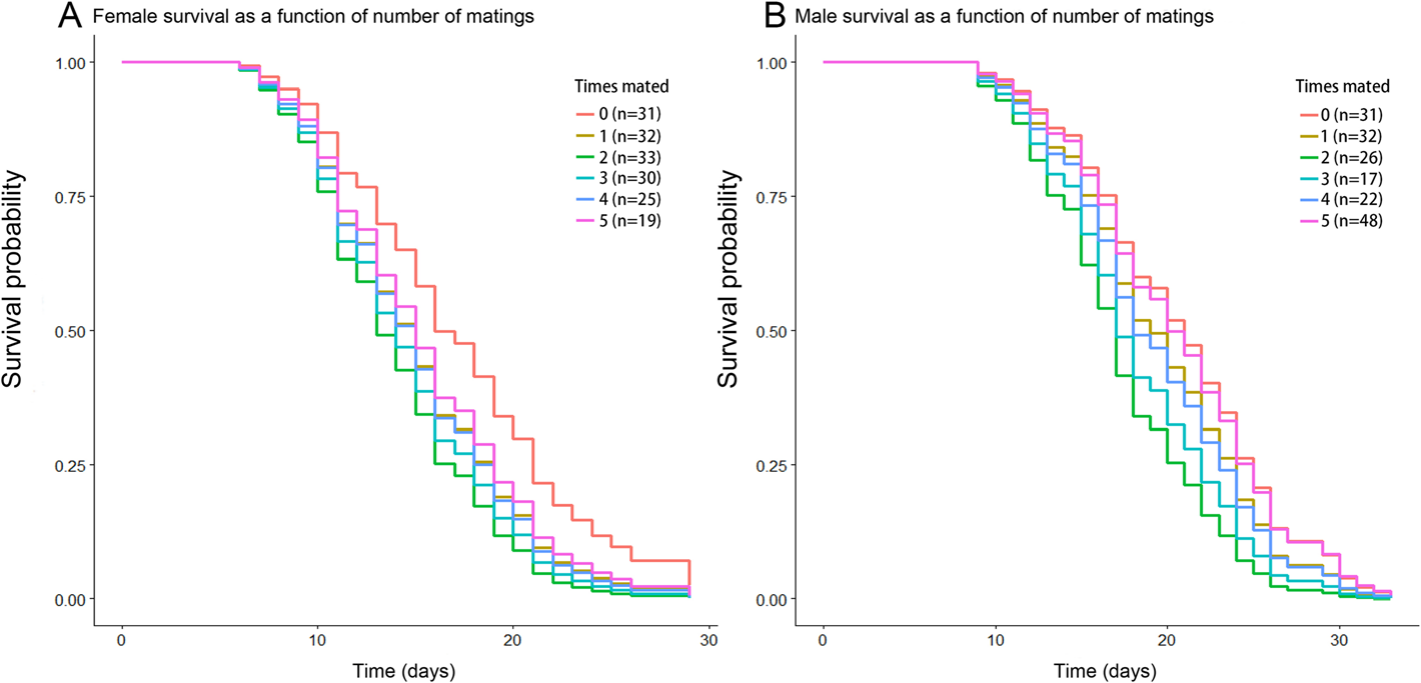
Longevity (in days) of females (**a**) and males (**b**) in relation to the number of matings. The number of matings did not affect the longevity of males (*z* = −1.696, *P* = 0.09) or females (*z* = − 1.379, *P* = 0.17)

Overall, females lived significantly shorter than males (Coxph: *χ*^*2*^ = 36.86, *df* = 1, *P* < 0.0001), but the number of matings did not affect the longevity of males (Coxph: *z* = − 1.696, *P* = 0.09) or females (Coxph: *z* = − 1.379, *P* = 0.17) (Fig. 1).

### The number of successful matings depends on mating history in both sexes

**Fig. 2:**
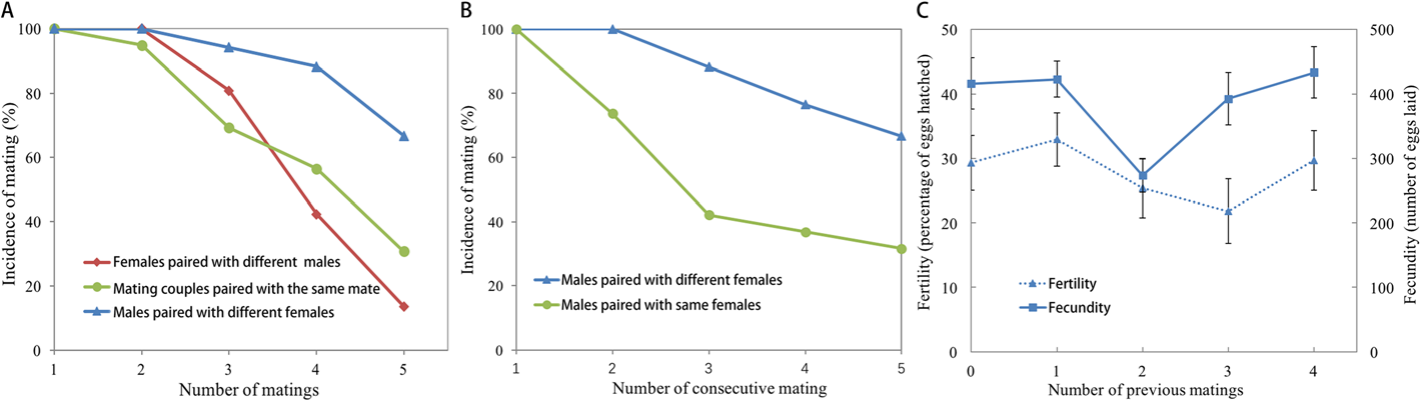
Mating frequencies and reproductive output. **a** Mating frequencies in repeated (green lines) and polygamous matings (blue lines: males, red lines: females). **b** Consecutive matings of males in repeated matings (green line) and polygamous matings (blue line). **c** Fertility (blue dashed line, ± SE) and fecundity (blue line, ± SE) of females related to the number of previous matings in their male partners in polygamous matings (treatment 3). Fecundity of females that mated with twice-mated males beforehand was significantly lower than females that mated with virgin males (*P* = 0.0199), females that mated with once-mated males beforehand (*P* = 0.0256), and females that mated with four times mated males beforehand (*P* = 0.0326)

Both sexes mated at least twice in the five experimental nights when offered different mates, and the number of successful matings declined with increasing mating nights in both males and females (Fig. 2). Analysis of a fitted *glm* indicated that the interaction term for sex and treatment was bordering significance (*χ*^*2*^ = 3.8097, *df* = 1, *P* = 0.051). Males mated significantly more than females when offered a different mate for five consecutive nights (Tukey *post-hoc* test: *P* = 0.036; Fig. 2a).

When classifying males by the number of times each male successfully mated (which was possible by checking the presence of a spermatophore in each female that was used; explained in detail in the Methods section), we found that 67% of the males mated in all five nights when paired with a new virgin female every night (polygamous matings) (Fig. 2a, blue line). In comparison, 81% of the females mated three times in five nights, but 42% of the females mated more than three times and only 14% of the females mated in all five nights (Fig. 2a, red line). The probability of mating five nights in a row with a different mate was significantly different between males and females (*G*-test with Yates correction; *G* = 39.9, *d.f.* = 1, *P* < 0.001). In the repeated matings, only 31% of the pairs mated in all five nights (Fig. 2a, green line). When counting the number of successful matings in consecutive nights, which is only possible in males as females would have to be dissected, all males successfully mated in two consecutive nights, while 88% of the males successfully mated in 3 consecutive nights and 77% of the males successfully mated in 4 consecutive nights in the polygamous matings (Fig. 2b). In the repeated matings, 74% of the males successfully mated in two consecutive nights, while 42% mated in 3 consecutive nights and 37% mated in 4 consecutive nights.

### Female reproductive output is affected by male mating history

The mating history of males did not affect the fertility of singly mated females (GLM: *χ*^*2*^ = 4.24, *df* = 4, *P* = 0.375), but it did affect their fecundity (GLM: *χ*^*2*^ = 13.31, *df* = 4, *P* = 0.01; Fig. 2c). Specifically, using Tukey contrasts we found that the fecundity of females that had mated with twice-mated males beforehand was significantly lower than females that mated with virgin males (Tukey *post-hoc* test: *P* = 0.023), females that mated with once-mated males beforehand (Tukey *post-hoc* test: *P* = 0.009), and females that mated with four times mated males beforehand (Tukey *post-hoc* test: *P* = 0.009) (Fig. 2c). When females were paired with a new virgin male every night, additional matings beyond 3 decreased their reproductive output, whereas this decrease was not apparent when females remated with the same male (i.e., in repeated matings). Corrected for the number of matings, females in the polygamous matings treatment produced significantly less offspring than females in the repeated matings treatment (*F*_1,84_ = 7.31, *P* = 0.008; Fig. S1a), while males produced similar numbers of offspring in the polygamous and repeated matings treatments (*F*_1,84_ = 1.29, *P* = 0.26) (Fig. S1b).

### Bateman gradient is different for males and females

**Fig. 3:**
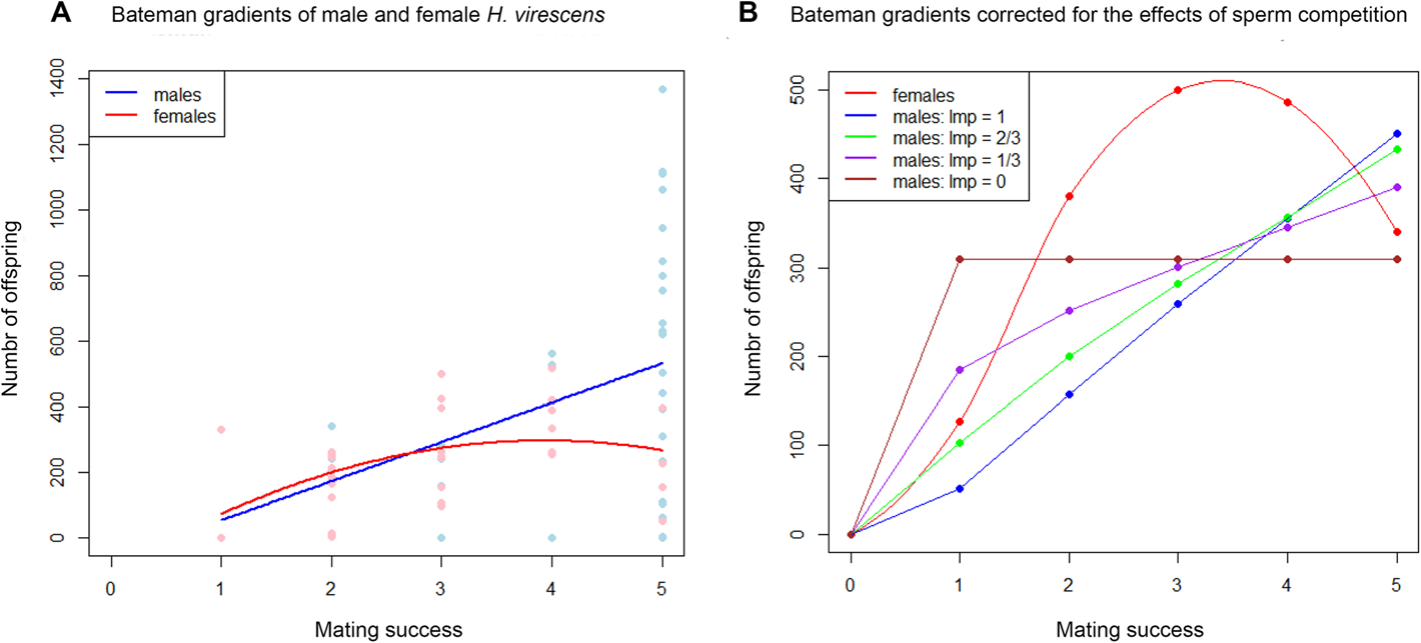
**a** Bateman gradients of males and females determined from the experimental data, **b** Bateman gradients determined by the simulation results, i.e. corrected for the effects of different last-male sperm precedence (LMP) scenarios. Blue line: LMP = 1, green line: LMP = 2/3, purple line: LMP = 1/3, brown line: LMP = 0

Male reproductive success was significantly and positively affected by the number of matings (*F*_1,35_ = 5.19, *P* = 0.029; Fig. 3a). A linear regression line best fit the data, each additional mating resulted in 119.7 ± 52.6 additional offspring (*R*^*2*^ = 0.10). For the females, a quadratic model fitted the data better (*F*_2,32_ = 3.49, *P* = 0.042, Fig. 3a) than a linear model (*R*^*2*^ = 0.13). This quadratic relationship suggests that the reproductive output of females increased with every additional mating until the females had mated 3 times, after which it decreased. The coefficients of variation (CV) in reproductive success was 0.82 (*n* = 37) for males and 0.55 for females (*n* = 36).

### Sperm precedence affects male Bateman gradient

As stated in the introduction, sperm precedence and paternity allocation studies in *H. virescens* have shown that sperm precedence ranges from 100% first or last male paternity to complete paternity mixing, which is similar to patterns found in other Lepidoptera [54–58]. Therefore, we theoretically investigated various sperm precedence scenarios by modeling the mating dynamics in a population of moths, using data from the mating experiments described above and available in the literature. Specifically, we assumed that mating probabilities were proportional to the available males and females in each night, that mating probability deterministically determines mating rate, and that 60% of the females that mated in one night were available for mating the next night, as this was found to be the mean percent in the “polygamous matings” treatment (see Fig. 2a). We also assumed a cumulative reproductive output for females as fitted by the statistical model on our experimental data (see Fig. 3a). Interestingly, in all sperm precedence scenarios, when comparing males and females with equal numbers of matings, the average reproductive output of males exceeds the reproductive output of females only when both sexes mate more than four times, except when last-male precedence is set to zero, i.e., when the first male mate sires all offspring of a female (Fig. 3b).

## Discussion

Our results show that multiple matings are beneficial as well as costly for both sexes in the polygamous moth species *H. virescens*, and that the costs and benefits depend on the mating history of the individuals and their mating partners, as we specify below.

### Remating is beneficial for both sexes at the start of the mating season

The positive Bateman gradient that we measured for both sexes of *H. virescens* up to three matings shows that multiple matings are in principle beneficial for both sexes. However, the fact that for females the measured relative number of offspring as a function of relative mating success fitted a quadratic model better than a linear model, which is traditionally used for Bateman gradients, indicates that the benefits for a females depends on her mating history; her reproductive output increases in the first three matings, which sets the stage for female-female competition whenever male reproductive output is increasing at a lower pace – which in part depends on the degree of last-male sperm precedence. However, after three matings the benefit for females to remate becomes negligible or even negative, while the benefit for males still increases. Despite these negligible benefits, females may still seek additional matings for other direct benefits that increase her number of offspring, for example to avoid sperm depletion [22], and/or to obtain matings with high (er) quality males that increases her reproductive output (e.g., [10, 17, 24, 59]), or for indirect benefits, e.g., to increase the genetic variation among her offspring [8]. Interestingly, the female Bateman gradient is similar to the male Bateman gradient, based on the measured data (Fig. 3a), but steeper than the male gradient when sperm precedence is taken into account (Fig. 3b), at least until she has mated three times. These results suggest that males are possibly the more choosy sex early in the mating season, while females likely become the more choosy sex later in the mating season.

### Multiple matings are costly for both sexes

Even though we found no significant differences in longevity between males and females that had mated once versus males and females that had mated multiple times, our results do suggest biologically relevant costs to multiple matings in both males and females. Specifically, in males the cost of producing ejaculates can limit the number of successful matings, which may explain our result that only 67% of males remated with a virgin female in all five consecutive nights (Fig. 2a). This may be due to the fact that a lepidopteran spermatophore can be 5–10% of the male’s body weight [42, 60]. In addition, the fact that we found female fecundity to be significantly lower when mated with males that had already mated twice suggests that these males produce a lower quality spermatophore. Our finding that only 31% of the males mated on five consecutive nights, when confined with the same female for five nights, may indicate that males decrease investment in a female that he has already mated with, i.e., sperm allocation trade-off between male investment in current versus future reproduction [22, 24, 60]. This behavioural phenomenon is commonly known as the Coolidge effect [61]. Of course, the reduced mating of males with the same female may also be due to a female’s change in behavior or physiology. For example, a female may decide not to remate with the same male, which she may recognize by the same ‘perfume’ that males apply with their hair pencils to the female during mating [43].

In females, our finding that 81% of the females mated three times in five nights, but only 42% of the females mated more than three times (Fig. 2a), is in accordance with the observation that females gain more offspring until three matings, while after three matings the reproductive output levels off (Fig. 3). The fact that only 14% of the females mate on five consecutive nights in polygamous matings implies some intrinsic, energetic cost for females to (re)mate. The cost of reproduction for females not only includes the production of eggs, but also the costs of female calling to attract a male. Even though the costs of female calling have generally been hypothesized to be negligible, recent findings do suggest some costs of female calling [48, 49].

### No benefits from mating with virgin mates

Males may gain higher reproductive output when mating with a virgin female than with a mated female, because Proshold et al. [47] found that ~ half of her eggs are oviposited after the first mating. However, the fact that we cannot discriminate between the Bateman gradients of males that mated with the same female and males that mated with new, virgin females on five consecutive nights (Fig. S1a) suggests that this effect is smaller than expected. One reason for this may be that females produce more offspring after multiple matings, but even more so when remating with the same male compared to mating with multiple males (Fig. S1b). For females, there seems to be no differential benefit for mating with virgin or mated males either, as we found no difference in fecundity between females mated with virgin or previously mated males (Fig. 2c). However, females did have higher reproductive output when mating consecutively with the same male (“repeated mating” treatment) than those mating with new virgin males in consecutive nights (“polygamous matings” treatment) (Fig. S1b), which resulted mainly from the fact that the reproductive output of females in the “polygamous matings” treatment decreased after remating with a new virgin male 4–5 times. This suggests that the spermatophores of males may contain extra nutrients for the females [60], but also (small amounts of) toxic substances, similar to the accessory gland proteins in *Drosophila melangaster* [62, 63]. These substances may only be toxic after a certain threshold, which in the case of *H. virescens* appears to be reached with 4 matings.

### Sperm precedence affects male Bateman gradient

Including male sperm competition in the Bateman analysis substantially affected the relative benefits of multiple mating for males. Sperm precedence determines the steepness of the Bateman curve for males and thus the relative gain of males to mate again (Fig. 3b). For example, in the extreme case of 100% last male sperm precedence, the average number of offspring increases much faster with every successful mating than in the case of 100% first-male sperm precedence (where males do not gain any paternity from additional matings after the first night). In *H. virescens*, the most likely paternity scenario is that roughly two thirds of the offspring is sired by the last male (green line in Fig. 3b). With such effects of sperm competition, males need to mate more often than females to reach similar reproductive success, biasing the OSR towards females, because males need to invest time and energy into new spermatophores for mating. Additionally, increased last-male paternity results in a steeper Bateman gradient for males (Fig. 3b) and thus increased variance between males with different mating histories.

## Conclusions

In conclusion, our results show that the costs and benefits depend on the mating history of the individuals and their mating partners. In light of who is likely to be the choosy sex, the sex with a steeper Bateman gradient indicates stronger directional selection for mating success and thus that sex predominantly competes for mating partners [7], while the other sex is likely the more choosy sex. As the female Bateman gradient is steeper than the male Bateman gradient until she has mated three times, males are possibly the more choosy sex early in the mating season, while females likely become the more choosy sex later in the mating season. As the steepness of the male Bateman gradient depends on the level of sperm precedence, sperm competition effects should be considered when comparing Bateman gradients for different sexes of a polygamous species to assess which is the more choosy sex. With these results, we conclude that in a polygamous species the choosing sex roles likely change dynamically with the mating status of males and females, depending on direct reproductive costs and benefits of multiple matings, and on sperm competition. Our life-history perspective on the mating dynamics of a polygamous moth species sheds new light on sexual selection for male and female sexual attraction.

## Methods

### Aim, design and setting of the study

To assess how sexual selection dynamics change over the course of a lifetime in a polygamous moth species, we investigated the direct reproductive costs and benefits of multiple matings, as well as the role of sperm competition in reproductive success. To identify the factors that may affect reproductive success, we used two different setups: repeated matings and polygamous matings, following [following 54] and described in detail below (experimental design). Using computer simulations, we also explored differences in absolute reproductive success between males and females. All experiments were conducted between November 2014 and February 2016 in the laboratory at the Institute for Biodiversity and Ecosystem Dynamics, University of Amsterdam.

### Description of materials

Populations of *H. virescens* moths have been laboratory reared at the Institute for Biodiversity and Ecosystem Dynamics, University of Amsterdam, since 2011. The rearings originate from North Carolina, where eggs were first collected from the field in 1988 [64], supplemented with new field collections over the years, and reared at the Max Planck Institute for Chemical Ecology from 2003 until 2011 (see also [65, 66]).

Larvae were reared individually on artificial pinto bean diet under controlled conditions in a climate chamber (60% relative humidity (RH); 25 degrees C; 14 h light: 10 h dark with lights off at 11.00 am). Pupae were checked daily for eclosion. Newly emerged adults were sexed and placed individually into a plastic cup (37 ml; Solo, Lake Forest, Illinois) with sugar water (cotton soaked with 10% sucrose). In all mating experiments, we used two- to three-day old virgin moths, and pairs were always from different families to avoid inbreeding effects. All experiments were conducted in clear plastic beakers (473 ml; Solo, Lake Forest, Illinois) covered with gauze, so that the moths could be observed but could not escape.

### Experimental design

We determined the longevity and lifetime fecundity and fertility of individual moths, making use of the experimental design that compares repeated matings with polygamous matings [67] to control for the factors partners and matings separately. Bateman gradient is defined as the slope of regression of the number of offspring on the number of mates [68]. To measure Bateman gradients, the data were combined from four treatments in our study. Specifically, four treatments were set up (see Table 1): a) one virgin male or female was placed per beaker and observed for five consecutive nights (treatment 1: no matings, females *n* = 30; males n = 30), b) virgin individuals were paired with a virgin mate on the first night, after which the sexes were separated into different beakers (treatment 2: mated once, females *n* = 29; males *n* = 31), c) individual moths were paired with a new, virgin mate every night for five consecutive nights (treatment 3: polygamous matings, females *n* = 59; males *n* = 63), d) individual moths were paired with the same mate every night (treatment 4: repeated matings, females *n* = 52; males *n* = 52) for five consecutive nights. To pair the individuals with a virgin mate every night, after each night the couples were separated into different beakers and given a new virgin mate. Individuals paired with different and same mates that died prior to the fifth night were excluded from the analyses (5 females and 2 males in treatment 3, four couples in treatment 4). Female fecundity was defined as the lifetime egg production of a female and female fertility was defined as the total percentage of eggs that hatched. Male fecundity and fertility was measured indirectly by counting the total number of eggs laid and the total percentage of eggs that hatched from his female partners.

Successful copulations were determined as follows. Moths are completely inactive during the daytime and become sexually active at specific hours at night [69]. *Heliothis virescens* is sexually active between three and 6–7 h after sunset [44, 45] and matings last on average 3 h [43]. Therefore, for five consecutive nights individual moths of all four treatments were observed every half hour with a red light to note copulating pairs, starting at the onset of the dark period (scotophase), in a climate chamber with the same conditions as described above. As spermatophores are formed during mating and remain in the bursa of the female, the number of successful matings can be determined by dissecting the female at the end of her life [37, 42]. To measure their longevity, all moths from the experiments were kept separately and fed with sugar water (cotton soaked with 10% sucrose) every 2 days until death. Therefore, after death adult females were dissected under a microscope to count the number of spermatophores inside each female, which was taken as the number of successful copulations.

### Effects of mating history

To assess the effects of successful matings with virgin partners or partners with different mating histories, we first determined the number of successful copulations, as described above, after which we assessed the successful mating incidences of males and females in treatments 3 and 4. To evaluate the effect of previous successful male mating history on the reproduction of singly mated females, only the males from the “polygamous matings” (treatment 3) were used, because in the “repeated matings” treatment we could not be sure in which night(s) a male had mated successfully with the paired female (i.e., when a mating resulted in a spermatophore) – we could only ascertain her cumulative mating status afterwards by dissection (see above).

To determine the fecundity of each moth, during the 5 days of the mating experiments, the eggs laid were collected and the moths were transferred into new beakers every day. After the experiments, all eggs from each cup were collected every second day and the number of eggs was counted using a grid under the microscope. Fecundity was calculated as the total number of eggs laid by each female during her life span. Fecundity of males could also be determined in this way, at least in treatment 3, by keeping track of each female that one male had mated with. In this way, we analyzed the fertility and fecundity of females and males that had mated with 0, 1, 2, 3 or 4 previously mated partners.

To determine the number of fertile eggs, the collected eggs were kept in separate cups (250 ml) until the eggs hatched. The number of hatched eggs (i.e., black eggs, which indicate developing larvae, and larvae) were counted 3 days after egg hatch started. Fertility was calculated as the percentage of eggs hatching.

### Reproductive success with sperm precedence

Using data from the mating experiments described above and available in the literature, we theoretically investigated various sperm precedence scenarios by modeling the mating dynamics in a population of moths through deterministic calculations. Specifically, we assumed that mating probabilities were proportional to the available males and females in each night, that mating probability deterministically determines mating rate, and that 60% of the females that mated in one night were available for mating the next night, as was found to be the case in the “polygamous matings” treatment (see Fig. 3b). We assumed relative cumulative reproductive output as a function of the number of matings as fitted for our data (Fig. 3a), and division of reproductive output over days since first mating as measured for singly mated females by Proshold et al. [47]. To determine the effect of remating on reproductive success for males, we used four paternity scenarios: a) 100% first-male sperm precedence: all offspring is sired by the first male a female mated with, and thus 0% is sired by any subsequent male, b) 0.33 last-male paternity: fertilization probability of sperm from earlier matings decreases by 33% each time a female mates with a new male, c) 0.66 last-male paternity, and d) 100% last-male sperm precedence: 100% of offspring is sired by the last male a female mated with. For females, we assumed that additional matings would result in more offspring as measured in the mating experiment, using the fitted relation between relative offspring production and relative mating success (see Fig. 3a and explanation below).

### Statistical analysis

All statistical analyses were performed in R software version 3.4.1 [70]. Survival analysis was conducted on the longevity data using Cox proportional hazards model (package: survival in R, [71]). The following covariates and their interactions were considered to be incorporated in these models: treatment, sex, number of matings. These models were simplified using analysis of deviance and AICc criteria. The model that was used to compare the longevity between males and females contained treatment, number of matings and sex without interactions as covariates. A separate Cox regression model was fitted for males and females to investigate the effect of multiple matings and treatment. These models contained treatment and the number of matings without interactions as covariates. To compare the effect of treatment on longevity, we used a Tukey *post-hoc* test at the 5% probability level for multiple comparisons (package: multcomp in R, [72]).

Separate Bateman curves were fitted for males and females. We regressed the number of live offspring on the number of matings (mating success) using linear models. To generate conventional Bateman curves that are comparable to results reported for other species, we only used the data obtained from treatment 3, i.e., individuals receiving a different virgin partner every night for five consecutive nights.

The effects of mating history on fecundity and fertility of singly mated females were analyzed using a generalized linear model (GLM) with a quasi-Poisson distribution (due to overdispersion), where either fecundity or fertility were the dependent variables and the number of previous matings as the independent variable. Tukey *post-hoc* tests at the 5% probability level for multiple comparisons were conducted to assess differences between previous male mating histories. To calculate the Coefficient of Variation (CV) in reproductive success, only the data of treatment 3 were used, where individuals received 5 virgin partners on five consecutive nights. The CV in reproductive success was calculated as the ratio of the standard deviation of the number of offspring and the mean number of offspring.

## Supplementary information

**Additional file :. Supplementary Figure S1.** Showing the Bateman gradients for males and females in the polygamous matings treatment (treatment 3) and the repeated matings (treatment 4).

## Data Availability

The data sets supporting the results of this article is available in Dryad, doi:10.5061/dryad.1vhhmgqq2.

## Abbreviations

Coxph: Cox proportional hazards regression model
GLM: Generalized Linear Model
LMP: Last Male Sperm Precedence
OSR: Operational Sex Ratio
