# Supplementary figures and images for: A life-history perspective on sexual selection in a polygamous species
Source: BMC Evol Biol. 2020 May 7;20:53. doi: 10.1186/s12862-020-01618-3 (PMC7206733; doi:10.1186/s12862-020-01618-3)

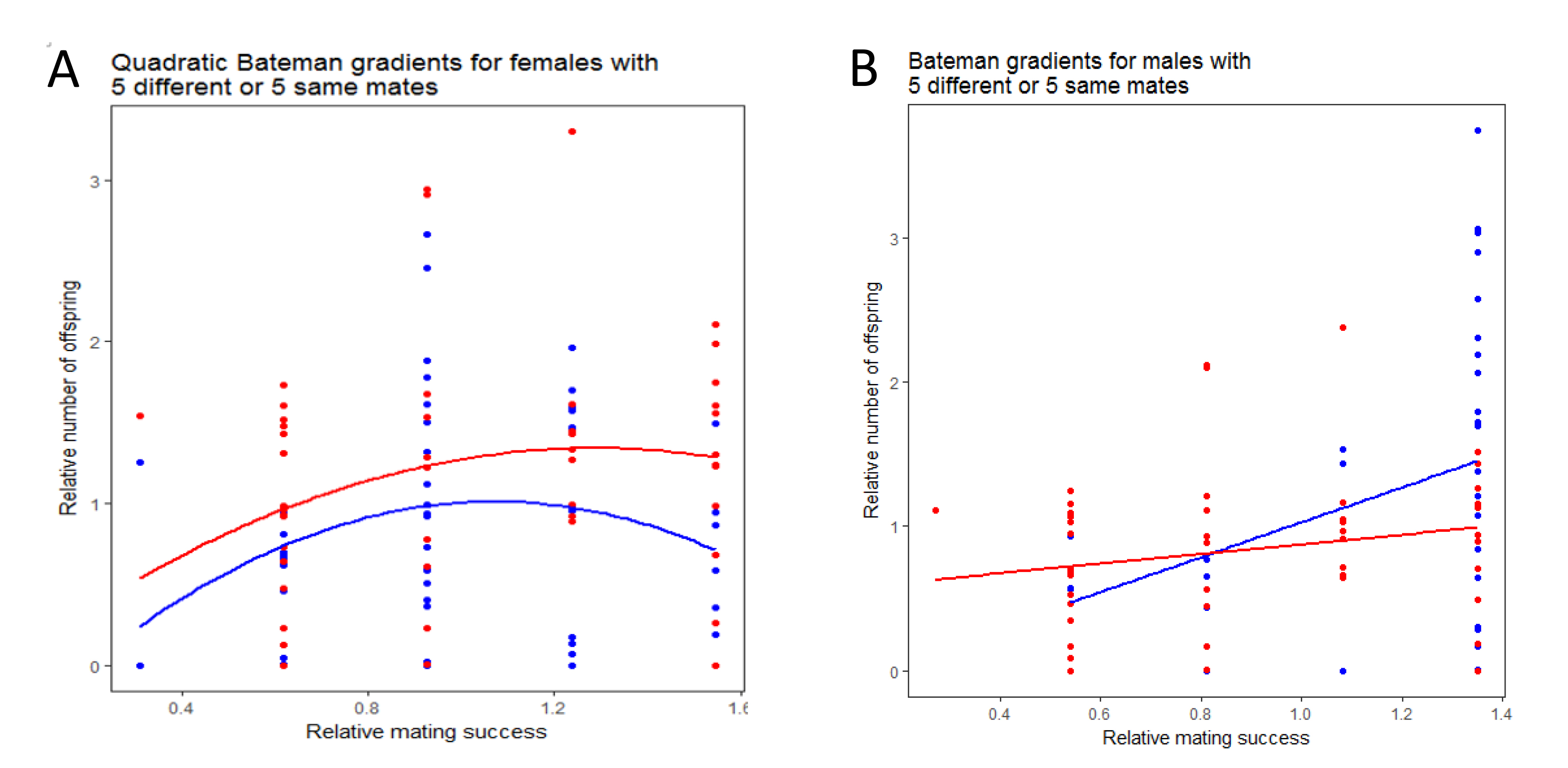

Supplement: Supplementary file 1 — Additional file :. Supplementary Figure S1. Showing the Bateman gradients for males and females in the polygamous matings treatment (treatment 3) and the repeated matings (treatment 4). [file 12862_2020_1618_MOESM1_ESM.jpg]
